# From Sensor Networks to Internet of Things. Bluetooth Low Energy, a Standard for This Evolution

**DOI:** 10.3390/s17020372

**Published:** 2017-02-14

**Authors:** Diego Hortelano, Teresa Olivares, M. Carmen Ruiz, Celia Garrido-Hidalgo, Vicente López

**Affiliations:** 1 Albacete Research Institute of Informatics, University of Castilla-La Mancha, 02071 Albacete, Spain; Celia.Garrido@uclm.es (C.G.-H.); Vicente.LCamacho@uclm.es (V.L.); 2 Faculty of Computer Science Engineering, University of Castilla-La Mancha, 02071 Albacete, Spain; Teresa.Olivares@uclm.es (T.O.); MCarmen.Ruiz@uclm.es (M.C.R.)

**Keywords:** bluetooth low energy, mesh topology, Industry 4.0, Collaborative Mesh, Internet of Things (IoT), sensor network

## Abstract

Current sensor networks need to be improved and updated to satisfy new essential requirements of the Internet of Things, where cutting-edge applications will appear. These requirements are: total coverage, zero fails (high performance), scalability and sustainability (hardware and software). We are going to evaluate Bluetooth Low Energy as wireless transmission technology and as the ideal candidate for these improvements, due to its low power consumption, its low cost radio chips and its ability to communicate with users directly, using their smartphones or smartbands. However, this technology is relatively recent, and standard network topologies are not able to fulfil its new requirements. To address these shortcomings, the implementation of other more flexible topologies (as the mesh topology) will be very interesting. After studying it in depth, we have identified certain weaknesses, for example, specific devices are needed to provide network scalability, and the need to choose between high performance or sustainability. In this paper, after presenting the studies carried out on these new technologies, we propose a new packet format and a new BLE mesh topology, with two different configurations: *Individual Mesh* and *Collaborative Mesh*. Our results show how this topology improves the scalability, sustainability, coverage and performance.

## 1. Our Previous Work

**Figure 1:**
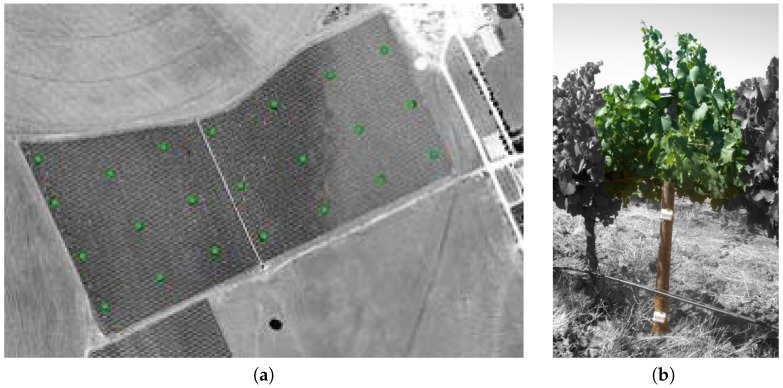
Full operational prototype of Wisevine Project. (**a**) Nodes location in the vineyard; (**b**) Placed devices.

Our research group at the Albacete Research Institute of Informatics (I3A) [1] has been actively involved in the study and deployment of wireless sensor networks (WSNs) for indoor and outdoor monitoring. Our research work started in 2005 with Wisevine [2], a regional Project with industrial partners. This Project enabled us to introduce this new technology into an important sector in our region: vine growing. We developed a computer-based information system and an operational prototype for capturing and processing data, which allows the data to be easily analysed by the specialists. Twenty two measurements points with three nodes located at three different heights were deployed (66 Mica2 nodes capturing data, see Figure 1). Data collected by the deployed sensors provides farmers with relevant information. This information can be used together with other tools for daily decision-making. Furthermore, the information generated throughout a season or year should prove valuable to improve farm performance.

**Figure 2:**
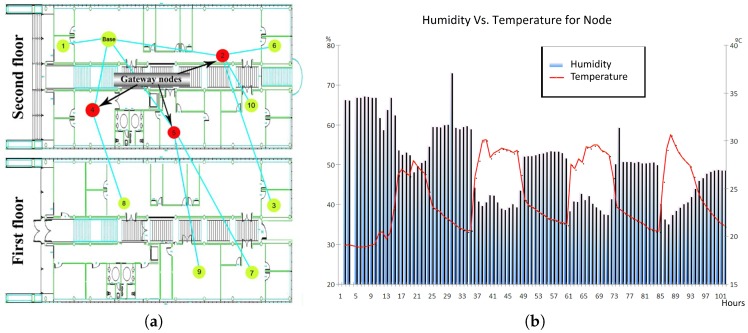
Experimental network of Intellbuilding Project. (**a**) Indoor network deployed; (**b**) Output example: humidity and temperature.

After this outdoor experience, we focused on designing and deploying WSNs for monitoring environmental indoor conditions, such as the temperature and humidity in an office space. Such a system should enable a quick and accurate diagnosis of the working environment: a must for productivity in a competitive society. The hardware equipment used to measure this information consisted of two development kits of micaZ nodes (MOTE-KIT2400) [3] (see Figure 2).

The discovered problems and the absence of a global architecture for WSNs took us to define ROBA (ROle-Based Architecture) [4], a new general purpose architecture for Wireless Sensor Networks. The main contributions of ROBA were its ease of use, simplicity, and versatility, since it fulfils the most important aspects in network configuration as physical design, medium access control, network management and application design. ROBA incorporated a role assignment module and harvesting techniques, using modern storage devices in combination with new power collection systems. The open design of ROBA allowed a large number of possible network configurations, combining different protocols, hardware and applications.

**Figure 3:**
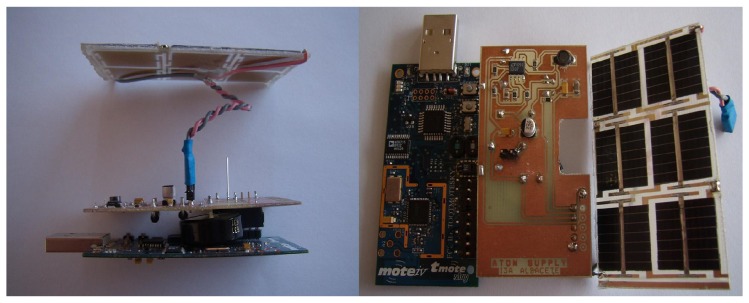
ATON I prototype.

We also developed ATON I [5] (see Figure 3), a battery less power management system particularly suitable for wireless sensor networks to be deployed outdoors. We ensured the long-lasting operation of a low-power sensor node adapting the duty cycle of the radio properly.

Two important protocols, Network and Media Access Control (MAC), were also developed [6]. NORIA [7], at network level, with a network auto-configuration mechanism making use of fuzzy logic rules, and SA-MAC [8], a power aware solution based on Time Division Multiple Access slot assignment. With SA-MAC a collision free communication tree is built in a previous network discovery phase and according to it, only in short periods of time the radio is turning on during the scheduling phase to establish communication, getting a large duty cycle and energy saving. The integration of these two protocols can be seen in [9,10].

An improved prototype of ATON I was published in [11], a power management framework incorporating a solar-powered batteryless power supply prototype, ATONII, which was properly coupled with improved SA-MAC, a power-aware MAC protocol whose operation is based on the dynamic adaptation of its duty cycle based on the microclimate conditions. Real world experiments showed the prototype works continuously in two extreme conditions: with a fixed and long duty cycle and with a short and variable duty cycle. We also implemented BANMAC [12], a collision-free MAC protocol for Body Area Networks that monitors and predicts the channel fluctuations and schedules transmissions opportunistically when the Received Signal Strength Indicator (RSSI) is likely to be higher. We presented experimental data which showed that the packet loss rate (PLR) of BANMAC is significantly lower compared to that of the IEEE 802.15.4 MAC.

**Figure 4:**
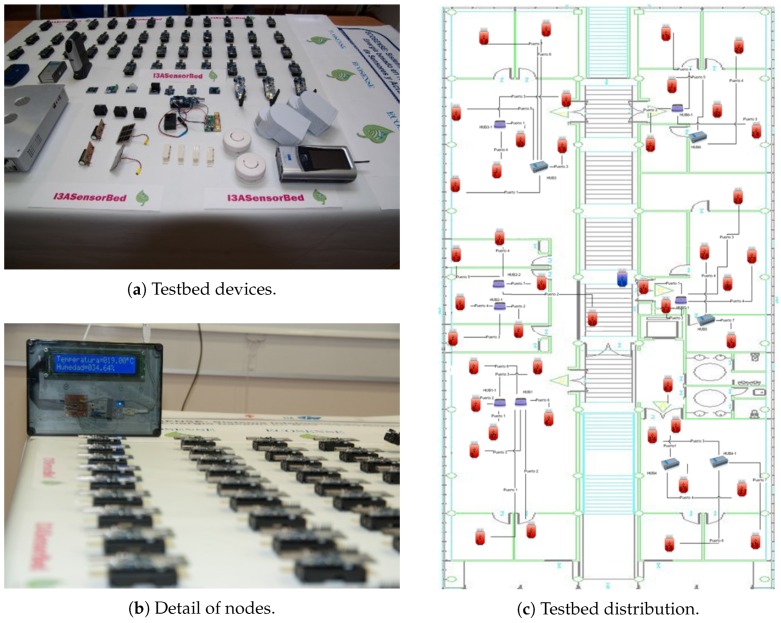
The I3ASensorBed.

Besides these publications, we carried out innovative projects, such as the aforementioned Wisevine and the Ecosense I (2008–2010) [13] and Ecosense II (2014–2017). The Ecosense I project achieved three objectives: to set up and deploy a wireless sensor network in our Research Institute in order to collect environmental indoor data (humidity, temperature, $CO_{2}$ level, luminosity), to set up a control and security system (presence control, doors and windows opening detectors) and to implement a testbed. We needed a real cabled sensor node platform in order to test our applications and protocols easily, and we implemented the I3ASensorbed [14] (see Figure 4), composed of 43 nodes deployed in the first floor of the I3A. These nodes incorporate temperature, humidity, $CO_{2}$, presence, smoke, door and window state (open/close) and energy consumption. In order to deploy all these nodes, the use of 12 USB hubs and 6 supernodes has been necessary.

## 2. Our Current Challenges

From this previous experience, with the current project Ecosense II we want to implement a reliable heterogeneous network to manage smart spaces, to identify multiples data sources and to define multiple experiments in real environments (smart homes and Industry 4.0 mainly) in the context of the Internet of Things (IoT).

IoT is a new paradigm that combines aspects and technologies coming from different approaches (ubiquitous and pervasive computing, Internet protocols, sensor networks, communication technologies, and embedded devices). The smart object is the core element of IoT. Smart objects are everyday objects able to collect environmental information and interact or control the physical world and, in addition, they can also be interconnected, to exchange data and information [15].

Furthermore, we are surrounding by different kinds of devices and applications gathering large amounts of data. Although smartphones remain leaders in the mobile industry, wearables and other innovative IoT devices are also having an unstoppable success. In this new reality, a problem arises; all these new devices do not use the same communication protocols so it is not possible to get all of them to work together. This fact has led us to face the challenge of improving the communications among different standards involved in the IoT revolution. Novel communication standards like Bluetooth Low Energy (BLE), also known as Bluetooth Smart (BS) [16] open enormous possibilities to communicate sensors, wearables, smartphones and smart objects with final users, enabling the utilization of network infrastructure to introduce or improve a wide variety of emerging applications.

Simultaneously, Industry 4.0 [17] or Industrial Internet is blooming and there is a long way to settle its standards. Industry 4.0 will allow the development and optimization of industrial infrastructure using new manufacturing practices which take advantage of Information and Communication Technology (ICT). However, the aim of this tendency is not only to introduce new technologies in industry but to connect and unify the different ICT components in a networked system. The modifications implemented in smart factories eliminate shortcomings, saving costs, and allow us to automate processes [18]. Energy efficiency in wireless communication protocols is a main requirement for use in the IoT. The BLE standard will become an important technology for the IoT due to its low power, low cost and small devices [19].

In [20], we defined an architecture for heterogeneous networks for easy management of smart spaces. This architecture can be used as reference to deploy real and flexible monitoring platforms based on IoT scenarios, making a large number of tasks easier for a user. We are working with innovative protocols such as BLE and smart network organizations [21].

In this paper, we show our current research line, which is focused on IoT and the study of standards to manage smart objects. BLE is presented as a technology for IoT and the new Industry 4.0 but this tendency brings new challenges for communication protocols. Different initiatives propose a new BLE topology: the mesh network topology. However, we have discovered that these initiatives have some weaknesses, therefore it is necessary to continue improving them. For this reason, we have proposed a novel BLE mesh topology with two configurations, which can be used for different use cases. Our proposal improves the network packet loss rate, without a substantial increase in the network traffic, which makes possible the network scalability.

**Figure 5:**
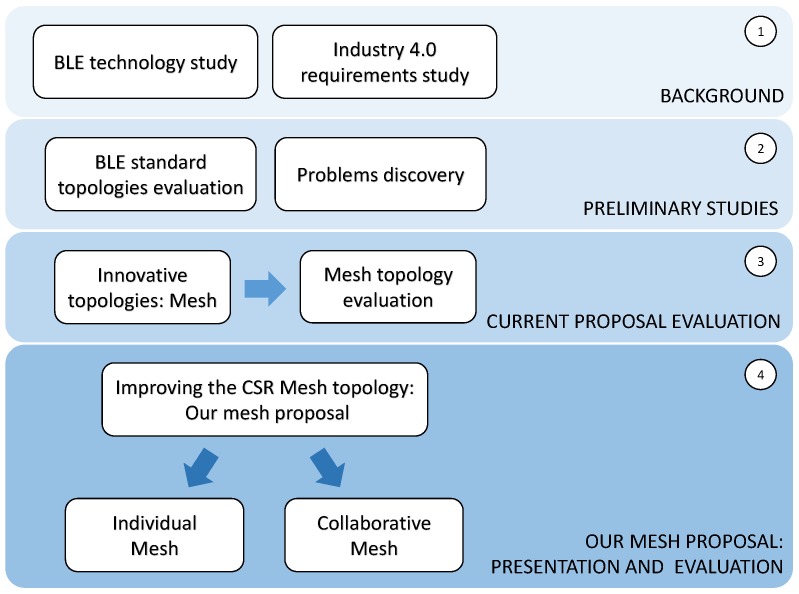
Paper road map.

The rest of this paper is organized as follows: Section 3 includes an overview of the works related with this paper; Section 4 introduces the BLE standard and its available topologies, as well as the new challenges for Industry 4.0; Section 5 presents a preliminary experiment to know if current BLE topologies overcome these challenges; in Section 6 we evaluate CSR Mesh in detail, the BLE mesh initiative, and expose its weaknesses by mean of different use cases. In Section 7, our mesh proposal is detailed and evaluated. Finally, conclusions and future works are shown in Section 8. To conclude this section and with the intention of making this work easily readable, Figure 5 shows an outline of the work flow presented in this paper.

## 3. Related Work

Although BLE is a very recent technology, and its standard has not a specification for a mesh network topology yet, there are interesting works analyzing the BLE standard and related applications: the Array of Things project of Chicago [22]; inter-vehicular communications [23]; power management in smart homes [19]; passengers control [24] or remote lock system [25]. However, our main focus is on papers related with mesh transmission using BLE. In [26], authors propose BLEMesh, a mesh network topology for BLE that takes advantage of the broadcasting capabilities of wireless networks. To reduce the number of packets retransmitted through the network, BLEMesh uses an opportunistic routing method. However, this proposal does not satisfy the Zero fails requirement of Industry 4.0. In [27] a light evaluation of a prototype of CSR mesh has been showed using the metric packet delivery ratio. After a not very clear study case, authors use the Octave simulator. They conclude that more studies are needed. In [28] authors implement a synchronization protocol. With this protocol nodes can not transmit in broadcast mode and then it will be impossible the free movement of the nodes such as smartphones, smartbands or different tools or instruments in the industry 4.0 environment.

On the other hand, the Bluetooth Special Interest Group (SIG) has officially announced the formation of the Bluetooth Smart Mesh Working Group, which will build the architecture for standardized mesh networking capability for Bluetooth Smart technology [29]. A new Bluetooth specification, Bluetooth 5 [30], has just been appeared, but there is no extra information about the mesh topology.

Well-known companies have also shown interest in incorporating this new functionality to BLE technology. The most important initiatives are the Nordic Semiconductor [31] and Cambridge Silicon Radio (CSR) [32]:

- The Nordic project [33] was created in collaboration with the Norwegian University of Science and Technology [34] as part of a thesis and is not part of the official SDK of Nordic Semiconductor. This is an open project, which uses the broadcast data transmission. This project uses Nordic series nRF51 [35] devices. These devices use BLE version 4.0, which causes packet loss in the mesh, because devices are not able to receive packets while they are retransmitting those already received.
- CSR is a company that has opted for the development of a mesh network topology for BLE. CSR has developed devices which use BLE version 4.1: the smart Bluetooth CSR101x family [36], as well as a proprietary protocol built on BLE, called CSRmesh [37]. This protocol allows to create a mesh topology by transmitting and receiving broadcast packets, like other projects.

However, mesh topology is not an exclusive BLE topology: ZigBee uses mesh topology to deploy low-power WSN [13,38], it is also well-known in Wi-Fi [39,40] and it allows connections among smartphones or tablets. However, BLE technology allows us to unify the advantages of both alternatives: to create low-power WSNs which are able to connect directly with users, using their smartphones.

This BLE network topology proposals open a new possibility in the Industry 4.0, because it will allow us to cover large areas completely. Thus, static or mobile (users) devices which are in the mesh can communicate with the mesh server or any other mesh device, regardless of their location.

## 4. Materials and Methods

BLE appeared in the Bluetooth specification 4.0 [41] in 2010 as a breakthrough technology for IoT. Moreover, BLE has seen an uncommonly rapid adoption rate, and the number of products designs that already include BLE puts it well ahead of other wireless technologies at the same point of time in their release cycles. This rapid growth of BLE is relatively easy to explain: BLE has gone further faster because its fate is so intimately tied to the phenomenal growth in smartphones, tablets, and mobile computing [16].

For the reasons described before, we selected BLE for detailed study and evaluation in real devices on our real deployed network, to check its strong points and weakness. In addition, Fourth Revolution Industrial has created new requirements for communication standards, and BLE may be an interesting option to manage indoor communications in future factories. In this section, BLE fundamentals are overviewed: available network topologies, and new requirements for BLE. Later, hardware devices used in our experiments are described.

### 4.1. Fundamentals

**Figure 6:**
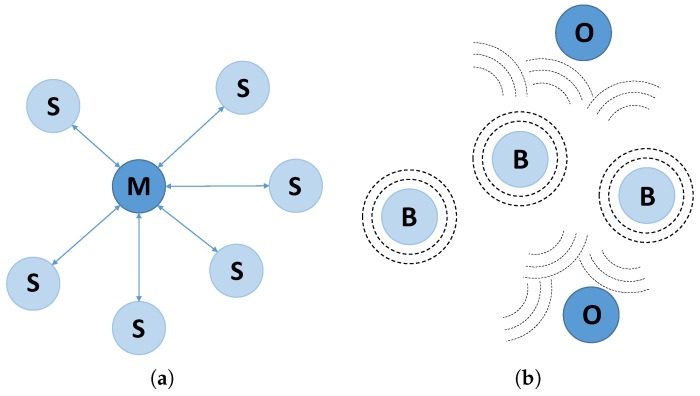
Available topologies in BLE standard (version 4.0). (**a**) Connection topology: (M) Master; (S) Slave; (**b**) Broadcast topology: (B) Broadcaster; (O) Observer.

In this section, we do not attempt to explain BLE in detail. We present the main characteristics necessary to facilitate the understanding of our work. The first BLE specification included two network topologies for data transmission: *connection* and *broadcast*, each one with its own advantages and limitations. BLE versions 4.1 [42], 4.2 [43] and 5.0 [30] maintain these topologies, which have been improved by combining different roles. However, these later versions are not implemented in most IoT devices. These two available topologies in BLE specification are described below:

- *Connection* topology: once the connection has been established, two BLE devices exchange packets in a permanent and periodical way. Two roles are used here: *master* (central) and *slave* (peripheral). A *master* device can connect with different *slave* devices, setting up a star-topology network (see Figure 6a). This operating mode allows the data exchange in both directions, between a *slave* device and the *master* device. In addition, a *master* device can use the *notification* and *indication* characteristics to read data immediately when they change. According to BLE specification, the number of slaves supported simultaneously by a master is unlimited. However, in real devices, with real memory limitations, this number falls to 4–8 (depending on the device limitations).
- *Broadcast* topology: a BLE device can transmit data using the BLE *advertising* mode to any BLE device in listening range, which uses the BLE *scanning* mode. This topology defines two roles: *broadcaster* (device which transmits data) and *observer* (device which receives data). In this case, data exchange is unidirectional, from the *broadcaster* to one or more *observers* (see Figure 6b).

These network topologies are enough to cover small and medium IoT installations. However, the recent emergence of Industry 4.0 includes IoT networks in factories, and changes the requirements of these networks. These new requirements are as follows [44,45]:

- Total coverage: the entire space of the building must be covered by the network, avoiding dead zones where the users can not communicate.
- Zero Fails: to provide a high performance, every transmitted packets must arrive at its destination, getting a success rate of 100% in communications and a PLR close to zero.
- Sustainability: covering both software (devices use efficient programs) and hardware (reducing the number of devices and using a low power standard). In this context, two new concepts appear: green-by (IoT network linking physical devices with operators to afford efficient operation) and green-in (techniques to encourage the deployment of cost efficient networks) [46,47,48].

### 4.2. Mesh Topologies in BLE Standard

Future industrial networks will enable a wide range of devices and services to be connected. So that there will be a need to collect as much real-time relevant data as possible. It is estimated that the quantity of connected devices will double or triple [45]. Then, the challenge will be connecting this large number of devices at the field level in a simple cost-efficient way. Of course demanding requirements for performance and reliability will still need to be met. Perhaps future networks with high numbers of devices should be hierarchical to simplify network management and operation. The star topologies have some advantages, such as lower latency and higher reliability, but also disadvantages mainly the failure of a central device will disconnect all attached devices. Thus, the use of more complex structures, such as extensively meshed network topologies, will increase. With the adoption of new protocols, these networks will need less management efforts and will offer quick network reconfiguration and service assurance [45].

As mentioned above, mesh network topology is not yet implemented in the BLE standard. Nevertheless, there are some initiatives which implement this topology over BLE. To explain in detail how this topology is implemented, we have focused on CSR mesh, because their devices use BLE version 4.1, which allows us to combine the different BLE roles.

CSR proposes the following mesh topology, built on BLE protocol, which uses three different BLE roles simultaneously:

- *Broadcaster*: CSR devices transmit data packets as broadcasters. These packets are coded and have a format which does not follow completely the BLE standard. To ensure that the packets arrive at the target device, the same packet is sent 3 times, the source device repeats 3 times the packet sending.
- *Observer*: using BLE version 4.1, CSR combines broadcast role with observer role. Thus, devices can receive packets from other devices at any time, even while they are transmitting their own packets. If received packets have the correct format and comply specific conditions, then they are repeated in broadcast again. This action increases the global coverage of the source device, and the network traffic.
- *Advertiser*: this role enables other devices to establish a master-slave connection. Being a partially closed protocol, CSR introduces new devices in their mesh using a direct connection to one of its nodes, which will work as a bridge between the new device and the rest of the mesh, it means, repeating the original data with the proper format.

Thus, mesh topology created by CSR uses a flood mesh protocol. Nevertheless, the main problem of this routing protocol is the high number of retransmissions [49], which we will try to reduce. For this aim, now we need to define two parameter which we will use in our study:

- Time To Live (TTL): one byte of the packet indicates the maximum number of times this packet can be retransmitted, or the maximum number of hops among devices possible. Each time a device receives and retransmit a packet, this number is decremented, discarding the packet if this byte is 0.
- Package ID: each of the packets transmitted by a device has an identifier, which will be the same while the packet remains in the network. This identifier allows devices to discard packets that have previously retransmitted, checking only this field.

### 4.3. Hardware Platform

This section shows the BLE devices used in our studies and experiments. We have chosen Waspmote devices from Libelium [50], because they can be equipped with multiple sensors and BLE 4.0 radio chips, and CSR1010 devices for evaluating their BLE 4.1 radio chips in mesh network topology.

**Figure 7:**
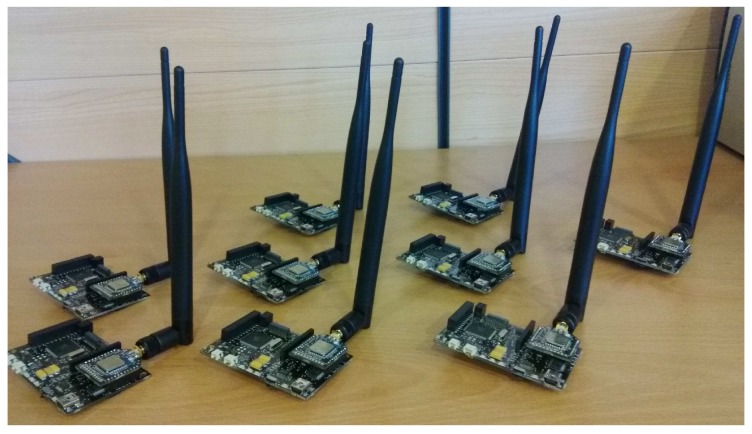
Waspmote devices equipped with BLE module.

Waspmote is a modular device (see Figure 7), which allows us to deploy WSNs using a large number of protocols for data transmission by means of different modules. Its specifications are: microcontroller ATmega1281 (14.7456 MHz); SRAM memory (8 KB), EEPROM (4 KB), FLASH (128 KB) and SD (2 GB); clock RTC (32 KHz); dimensions of 73.5 × 51 × 13 mm (L × W × H) and weight of 20 gr; and finally, different operation levels to reduce the consumption: *ON* (15 mA), *Sleep* (55 μA), *Deep Sleep* (55 μA) or *Hibernate* (0.07 μA). Libelium provides us with multiple options to choose a wireless communication protocol. Specifications of the BLE radio module are: chipset BLE112 from BlueGiga [51]; RX sensibility of −103 dBm; TX Power interval between −23 dBm and +3 dBm; antenna of 5 dBi; security using AES-128; range of 100 m; consumption modes: sleep (0.4 μA)/RX (8 mA)/TX (36 mA).

**Figure 8:**
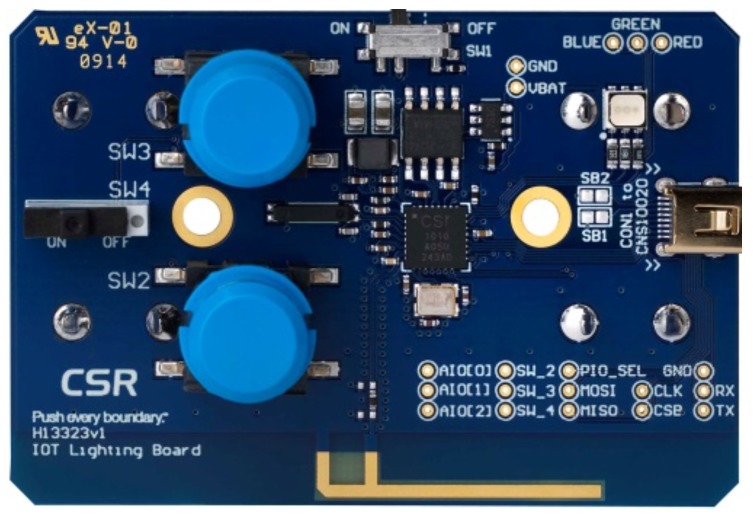
CSR1010 device [36].

CSR1010 devices (see Figure 8) have a BLE 4.1 radio with direct single-ended 50 Ω antenna connection, which allows them to transmit BLE data in any direction, at a distance between 20 and 30 m depending on existing obstacles; a 16-bit microprocessor with 64 Kbytes RAM and 64 Kbytes ROM; 1 μA integrated key scanning hardware; PWMs and quadrature decoders; peripheral I2C and SPI (debug); analog IOs and UART interface; up to 32 re-assignable programmable digital IOs; up to 4.4 V direct supply connection for Li-poly batteries.

## 5. Preliminary Evaluation: BLE Standard Topologies for Industry 4.0

A preliminary study has been carry out to determine if the available network topologies are suitable for new Industry 4.0 challenge. For this study, Waspmote devices with a BLE radio module have been used to deploy our BLE network.

**Figure 9:**
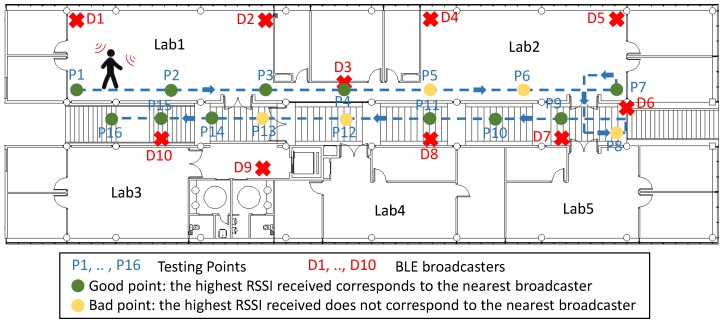
Results obtained in preliminary test to check the performance in a BLE IoT installation.

Thus, a 10-BLE-device network was deployed in our institute, I3A. For data transmissions, devices use the broadcasting capability: the static BLE devices transmit packets as *broadcasters*, and an user equipped with a mobile device (which is a *observer*) receives these packets in different points while he is moving through the building (from P1 to P16 in Figure 9). Measurements relating to the number of packets and their RSSI have been taken in different points of the walk-through of the user to know if the deployed network works correctly. Figure 9 shows the situation of the static *BLE broadcasters* (D1,...,D10) and *Testing Points* (P1,...,P16). It has also been shown what we have called *Good Points* and *Bad Points*. In *Good Points*, the greatest RSSI corresponds to packets from the nearest device; *Bad Points* show both, where the higher RSSI received packets do not come from the nearest device, or the observer has not received any packet from this nearest static device.

As Figure 9 shows, only 68.75% of the testing points are *Good Points*, where the observer received packets from the nearest static device correctly. These results leads us to conclude that this topology does not fulfil the requirements of the Industry 4.0 identified above: there are several dead zones where there is not network coverage, and the PLR is too high.

Furthermore, *connection* topology has been dismissed due to the restriction in the number of slaves connected simultaneously to a master (this number depends on the memory of device, and, in practice, this number is 8 at maximum), and the impossibility of deploying a network hierarchy by means of connecting a master with other master devices working as slave (this characteristic was included in the specification 4.1 of Bluetooth [42], but most devices do not implement it). In most Industry 4.0 use cases, just 8 slave devices connected to a master device are not enough, because this is a very static and limited option, due to every slave device should be within the master slave range. In addition, these limitations affect the coverage of the network, and make hard to scale the network, which has a negative impact in its sustainability.

Once the current topologies specified in the Bluetooth standard have been ruled out for use in Industry 4.0 due to it does not meet its requirements, a new BLE network topology is necessary: mesh topology. As seen before, there are already some initiatives to use this topology in BLE technology. However, these first versions have weaknesses which are necessary to solve. So that, in following sections, we will present our new topology proposal. Moreover, it will be compared with the current initiatives, evaluating its performance and demonstrating that its packet loss is lower.

## 6. Discussion

This section is related in the following way: firstly, CSR devices and CSR Mesh are evaluated; secondly, problems found in this evaluation are established. In this way, the study cases are:

- Study of PLR in CSR devices. One of the most important requirements in Industry 4.0 is the *Zero fails* objective, to provide a high performance in networks, so this rate has a great impact. For this reason, to know the PLR for these devices in optimal conditions is necessary.
- Coverage study for CSR devices in real environments, which allows us to deploy a real network in a real environment.
- CSR Mesh evaluation in a real environment.

### 6.1. Packet Loss Rate in CSR Devices

As described above, packets are retransmitted three times in the CSR Mesh as a reliability measure and to ensure that the transmitted packets are received in different devices. When a device receives a packet, it retransmits this packet three times if it is not the destination device. If the same packet is received more than once, it is discarded and no action is taken. We have carried out a study to verify if this way of working is enough to provide the needed performance.

A simple mesh network was deployed for this experiment. This network had a single broadcaster device and a single observer device (CSR1010). The broadcaster sent packets to the observer. The study has been carried out by evaluating the PLR according to the number of repetitions of each packet. A new packet was transmitted each 5 s for 3000 s. If the repetitions were configured in broadcaster, a new packet and its repetitions were transmitted each 5 s. This experiment tried to evaluate the impact of this measurement on the network, so that the devices were in optimal conditions for transmitting and receiving data: in a direct line of sight and separated 50 cm, minimizing the packet loss due to distance or obstacles.

Table 1 shows the PLR for packets transmitted once, twice or three times. These results reflect an improvement when the number of transmitted packets grows. A PLR of approximately 16% was observed for packets transmitted only once; 3% when packets were repeated twice and 0% when the number of repeated packets was three. The need of transmitting three times is due to the fact that the BLE radio is not only used to receive packets, it has also to transmit advertisement packets (i.e., other devices can use this device like a bridge) and moreover, the radio must retransmit the received packets.

As this basic experiment shows, the operating mode taken by CSR mesh topology based on repeating the packets 3 times ensures the arrival of all packets, but increases the traffic of the network, the power consumption and the packet collisions in a saturated wireless frequency band. It could trigger a scalability problem when the number of devices in the network increases.

A new experiment was carried out to evaluate these new problems in a real network. In this new experiment, a network was deployed using a higher number of network devices to cover a real building, emulating a factory. However, before the network deployment, a coverage study was needed.

### 6.2. Coverage Study for CSR Devices

When deploying a real wireless sensor network, the first step is to carry out a coverage study to determine the least number of needed devices to cover the largest possible area with the lowest cost. For this reason, this coverage study was performed in the first floor of our research institute (48 m in length and 15 m in width).

**Figure 10:**
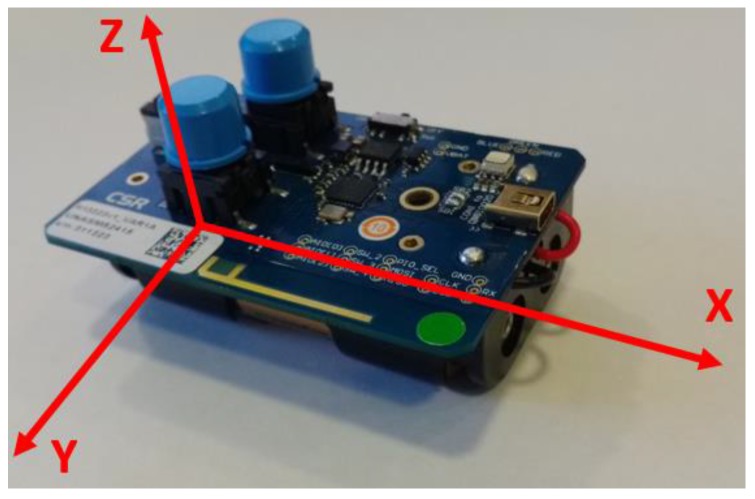
X, Y and Z plans in a CSR1010 device.

An important consideration when we want to evaluate the range of a device is the antenna. CSR1010 devices have an Inverted-F Antenna [52]. The radiation pattern in this antenna is circular in the XZ plane. So, we can distinguish two different area according to the device placing: the coverage range is favourable in the XZ plane (see Figure 10) and unfavourable in the rest of the plans.

In open areas with a direct line sight, the coverage range of CSR1010 devices is around 30 m. However, this range is lower in inside environments with obstacles, so it was necessary a study to evaluate this range in our laboratory.

In this experiment, two devices were used: a broadcaster placed 4 m from a observer, with constructive elements between them (see Table 2). This study is not used for any building, it means, it must be repeated for any building to ensure the minimum number of devices needed to cover its own area. In our experimental scenarios, 80 RSSI samples were taken in coexistence with other wireless technologies (Wi-Fi and ZigBee).

For each evaluated scenario, two experiments were carried out, regarding the device position: favourable and unfavourable position. The obtained results are shown in Table 2.

Using this previous evaluation, we were able to study how different construction elements affect the coverage range. For this purpose, we deployed a mesh network, avoiding the problematic elements, like columns, walls and particular storage racks.

We begun deploying a 10-CSR device network. Of course, all coverage area was covered, but our aim was to know the minimum number of devices needed to cover it. So that, the number of devices was gradually decreased up to we found that two devices were not able to cover the whole area. Therefore, we established that the minimum needed devices to cover this area was three.

### 6.3. CSR Mesh Evaluation

**Figure 11:**
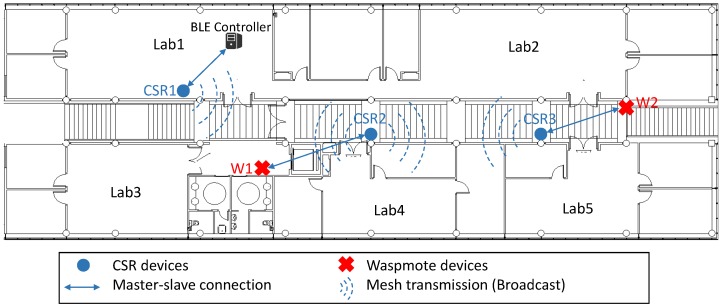
Network deployed using 2 sensor devices and CSR mesh topology.

Once we studied the behaviour of a single CSR device in optimal conditions, we wanted to know how really a CSR Mesh network works. For this purpose, the following steps were followed:

- By the previous coverage study, three CSR devices are needed to cover the first floor of our research Institute
- To guarantee a test environment as real as possible, we emulated an industrial environment where it was necessary to measure different parameters which were processed by a BLE controller. To do this, two Waspmote devices equipped with sensors were added into the mesh, using CSR devices as a bridge (following CSR topology). These Waspmote devices transmitted sensor data packet every five seconds. In addition, a BLE controller which receives, processes and stores BLE packets, was also introduced using the remaining CSR device. Figure 11 shows the final arrangement of the devices.
- In addition, to determine the influence of repeating packets on a real mesh network, CSR devices were configured with two different settings: transmitting each packet once, and transmitting three times (default mode).
- Finally, for each of the proposed configurations, the PLR was measured, as well as the number of packets received in each CSR device per second, to evaluate the packet traffic in network.

Table 3 and Table 4 show the network traffic (sent and received packets, respectively, by different devices). As a result of the mesh network topology, the broadcast mode and the proprietary code of the CSR devices, a typical traffic matrix is not available. Table 3 shows the number of packets sent by Waspmote devices (W1 and W2) and the number of these packets received by the BLE server, for two different network configuration. It can be observed that the number of packet repetitions is especially relevant for packets from W2, which have to be retransmitted by three different devices (CSR3, CSR2 and CSR1) and finally received by the BLE server. On the contrary, for packets from W1, which were retransmitted from CSR2 to CSR1, there are no significant differences when changing the configuration.

Table 4 shows the number of packets received by different CSR devices, as well as BLE server. From these results, important information has been obtained. Firstly, the number of received packets grows when the number of repetitions is higher. In other words, the network traffic is higher when CSR devices transmit each packet three times. Secondly, CSR1 device performed an important packet filtering, since only 42% of packets for the first configuration and 31% of packets for the second configuration are original packets, i.e., not repeated packets. Therefore, this connection will be maintained in following evaluations. Thirdly, there is an increase in the number of packets received by CSR3 device higher than the increase in the number of packets received by CSR2. It is quite the opposite to what is expected since the CSR2 device is located in the middle of the mesh network, so that it must receive packets from all the rest nodes in the mesh. This behaviour is unsuitable and ends up in troubles when the network has a high number of devices.

In Table 5 it can be observed the PLR of each sensor node, which are the source of the information. In the first configuration, CSR devices retransmitted each packet once, while in second configuration CSR devices repeated each packet three times. These packets could contain different data (including key data), therefore avoiding their loss can be crucial. The first conclusion we can draw is quite expected the results show a lower packet loss when CSR devices repeat each packet three times instead of one (3.21% against 16.73% on average). The second conclusion is related to the device positioning: the Waspmote 1 results are much better than the Waspmote 2 results due to the second one is farther from BLE controller (see positioning in Figure 11), so that, its packets need to be retransmitted by more devices (increasing their network hops).

As stated above, an important parameter of this type of network is the number of packets which are moved by devices at any given time (network traffic). Therefore, the number of packets received in the CSR devices were measured. These packets could be repeated (either a repetition from the same device, or a retransmission from another device), so that, the CSR devices must filter them and remove the duplicate ones. Referring to this, Table 6 shows the number of packets per second in each CSR device, for two possible configurations: CSR devices retransmit each packet once or three times.

In the Table 6 we can see the payment required to achieve that low PLR (see Table 5): each CSR device received 0,57 more packets per second on average when they repeated the received packets three times. Although it may not seem too much, it can make the situation unsustainable if the number of devices in the network is increased (in this case, the number of sensor devices was only 2, and the number of total network devices was 6).

As seen from this experiment, the PLR in CSR Mesh is acceptable, although for this acceptable PLR packets must be retransmitted three times. In addition, CSR Mesh is a proprietary protocol built on BLE, and scalability problems arise when different BLE devices are included in network, because they need a CSR device working as slave to bridging them with the rest of the network. The maximum number of non-CSR devices on mesh network is the number of CSR devices in it. Moreover, if a wider coverage range is needed, more CSR devices must be included. To solve these scalability problems, we propose some improvements. In our proposal, any BLE device with the observer and broadcaster roles, could take part in the network, and no bridge device is necessary. In next section, this proposal is detailed, as well as some experiments which were carried out to evaluate its packet loss and its network traffic.

## 7. Improving the CSR Mesh

As already described, one of the weaknesses of the CSR mesh network is its scalability, especially when new sensor devices are adding to it: to introduce a new non-CSR devices on network, the use of a CSR device as bridge is necessary. Because of great impact that BLE mesh network topology can have on the Industry 4.0, this network scalability must be complete, irrespective of the devices which conform the network. After the study of the behaviour of the CSR mesh, we decided to develop a new mesh proposal based on it. While to connect an external device to a bridge CSR device using a master-slave connection is necessary in the CSR mesh to take part of this network, our proposal removes this restriction. In this way, all BLE devices could broadcast packets with the specified format, which are retransmitted to their destination.

Although the behaviour of the devices may seem similar, there are important differences between the protocol proposed in this section and CSR Mesh, namely:

- Our proposal is an open protocol, in contrast to CSR Mesh. It allows us to implement our protocol in any BLE device. In addition, this characteristic will be very useful for future evaluations.
- Bridge devices are not needed. In our proposal, any BLE device can take part of the mesh network, because all nodes broadcast their data packets to their neighbours. It improves the scalability and the cost savings, due to bridge devices are not needed for each new added device in the network.
- Master-slave connection is not required in our proposal. It greatly improves the inclusion of mobile devices (like users smartphones). In this way, a user could move freely within mesh network, without worrying about master-slave connection interruptions.

**Figure 12:**
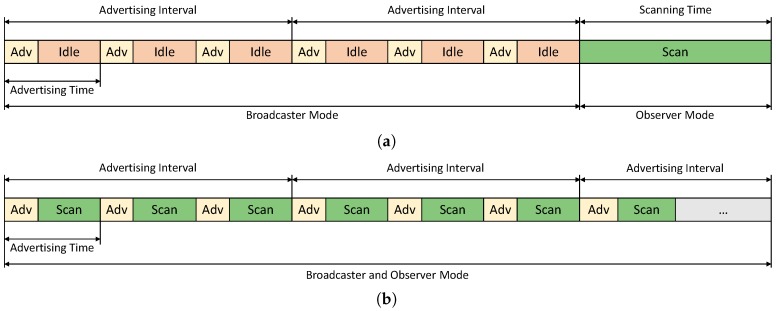
Differences between Advertising and Scanning processes in BLE version 4.0 and 4.1. (**a**) Advertising and Scanning processes in BLE 4.0; (**b**) Advertising and Scanning processes in BLE 4.1.

**Figure 13:**
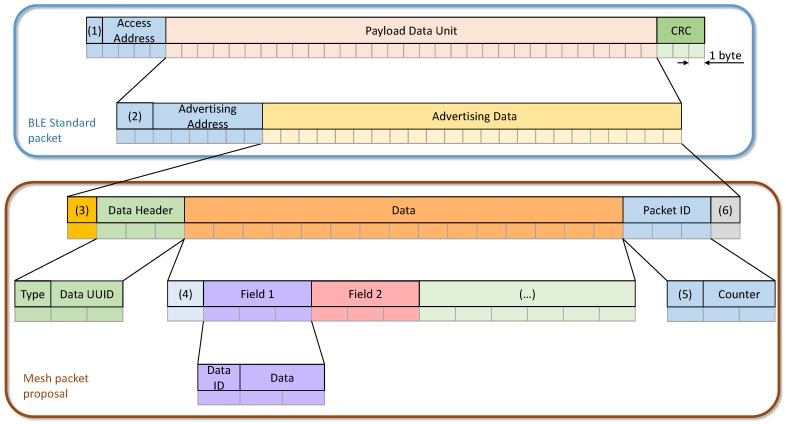
Our proposed mesh packet format. (1) Preamble; (2) BLE Header; (3) Advertising Data Length; (4) Destination Device ID; (5) Source Device ID; (6) TTL.

In addition to these important differences, for our mesh proposal, a new packet format was defined. This packet format is compatible with CSR devices, so that these devices can be included in our mesh as a backbone of it, since their BLE 4.1 chips allow us to obtain a better performance in this use case than if we used only BLE 4.0 chips. This better performance is due to the possibility of using observer and broadcaster modes simultaneously, without the need to switch them. A single BLE 4.0 radio only can transmit or receive data at a certain point. However, using BLE 4.1, to change the mode of operation from observer to broadcaster (or vice versa) is not necessary. It allows us to take advantage during the idle periods for doing other useful actions. Figure 12 shows these differences between BLE version 4.0 and 4.1:

As said above, we created our own packet format since CSR uses its proprietary packet format for its proprietary mesh protocol, and it is not available for users. However, in our proposal, we have developed our packet format following BLE standard, and it is compatible with CSR devices, to give the choice of including them in the network. Thereby, any two devices can communicate using the mesh when both of them are inside its coverage range. In this way, devices only have to transmit an advertising package, following the defined format, in their broadcaster mode to transmit data. Figure 13 shows our proposed format, which can be used by any developer to create a new mesh network. This packet format follows the BLE specification, so that, the most of its fields can be consulted in [41]. These fields are shown below:

- Preamble: all BLE packets have an eight bit preamble, which is used in the receiver for frequency synchronization, symbol timing estimation, and Automatic Gain Control training tasks. In advertising packets, as in this case, preamble must be 0xAA.
- Access Address: in advertising packets, access address is a 32-bit value, and for advertising packets shall be 0x8E89BED6.
- Payload Data Unit (PDU): packets have a variable size payload, from 12 to 37 bytes, which includes:
  – Header: a 16-bit header, where PDU type is specified. This PDU type is *non connectable undirected advertising*, which is used in broadcast data transmissions.
  – Advertising Address: this 6-octet field contains the BLE address. In our proposal this is a random address generated by device.
  – Advertising Data: this variable size field (from 4 to 31 bytes) contains data collected from sensors or device information (battery level, for example). Advertising Data contains the following fields:
    ∗ Advertising Data Packet Length: following the BLE standard, first octet contains the PDU length.
    ∗ Data header: contains two different fields. The first field is *Type* which indicates the PDU service. For mesh packets, its value is 0x16 (service data). The second field is a 16-bit *UUID*. To ensure the compatibility with CSR devices, the UUID for his packets shall be the CSR UUID.
    ∗ Destination Device ID: due to the use of random address, devices use an ID to identify them, which is shorter than a BLE address.
    ∗ Data Fields: are divide in two sections, an ID which indicates the data type and its length, and the data itself.
    ∗ Packet ID: each packet contains its own ID, to avoid the uncontrolled packet retransmission, this field includes the source device ID and a packet counter, to ensure the uniqueness of the packet at any given time.
    ∗ TTL: where TTL value which limits the lifespan of the packet is stored.
- Cyclic Redundancy Check (CRC): at the end of every packet there is a 24-bit CRC, which shall be calculated over the PDU.

Using our defined format, to transmit a packet to a device it is enough to know its identifier. This identifier device can be programmed in the memory of the device or be assigned dynamically by a BLE network controller.

In addition, this packet format provides two possible configurations for sensor devices, since it is possible to communicate with a mesh device without being a complete mesh device:

- *Individual Mesh*: sensor devices only transmit their data packets, but they do not retransmit the packets of other devices. This option provides us a lower traffic network.
- *Collaborative Mesh*: sensor devices transmit their data packets and they retransmit the received packet for other devices. This option increases the network coverage, but also the network traffic. Furthermore, to deploy a mesh network with this option using only sensor devices is possible, without the use of CSR devices.

Finally, all these modifications allow us to obtain different improvements:

- To know the source and destination device of each packet.
- To increase the size of the data field in each package.
- To introduce new devices working as mesh devices, either to transmit only their own data or to retransmit the received packets, without using a CSR device as a bridge. This advantage is notably accentuated when the devices we want to include in the mesh are mobile or wearable devices from users: smartphones, tablets, smartwatchs or smartbands, for example. While in the option proposed by CSR a device must establish a master-slave connection that will be lost when the user leaves the range of coverage of the bridge device, our option allows us to transmit packets to the network in general, accentuating the concept of mesh and total coverage.
- To add new devices, no matter how many bridge devices are available, since they are no required. In this way, our network provides increased scalability, sustainability, and saving cost, because we can eliminate CSR bridge devices, reducing the final number of network devices without compromising the network performance.

### 7.1. Evaluation of Our New Mesh Proposals

After the description of our mesh topology proposal, an in-depth study about its behaviour is necessary. In this study, we took advantage of the scalability of our proposed mesh and the possibility to include any type of device. So that, we used 8 Waspmote devices, 3 CSR devices and a BLE controller. In addition, Waspmote devices were configured in the two settings before defined: *Individual Mesh* and *Collaborative Mesh*.

**Figure 14:**
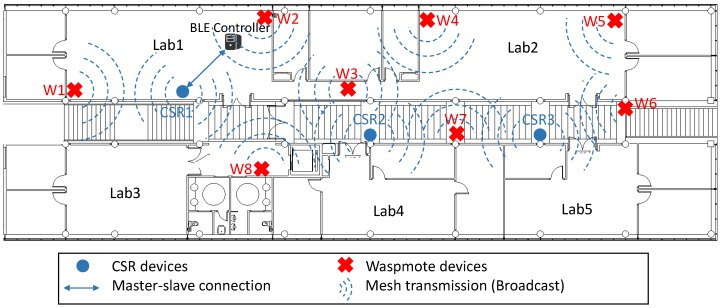
Network deployed using 8 sensor devices without master-slave connection.

To evaluate the behaviour of our mesh topology proposal and its scalability, these steps have been followed:

- Three CSR devices were deployed following the previous coverage study.
- As in previous experiments, an industrial environment was emulated where different parameters were measured. A BLE controller was included in the network using a master-slave connection (CSR1-BLE controller) to take advantage of the BLE 4.1 radio used by CSR devices. Moreover, in this experiment, 8 Waspmote devices equipped with sensors were included. These devices monitored different parameters, and transmitted their data each 5 s. Figure 14 shows the deployed network.
- Network configurations have been defined: *Individual Mesh* and *Collaborative Mesh*. For each configuration, the PLR for each Waspmote device and the number of packets per second received by each CSR was measured.

In the following sections, the behaviour of both configurations has been detailed. Moreover, for each configuration, a study for *Low Network Load* and *High Network Load* were carried out. On the one hand, using a *Low Network Load*, Waspmote devices repeat each packet once, which reduces the network traffic and the network PLR. On the other hand, using a *High Network Load*, Waspmote devices repeat each packet three times, which increases the network PLR and the network traffic. To choose the best option, the following studies were carried out.

### 7.2. Individual Mesh Evaluation

In this section we evaluate the first Waspmote device configuration: *Individual Mesh*. As stated before, using this configuration, Waspmote devices transmit their data packets using our mesh packet format and receive packets from others devices; if this device is the destination, it process the packet; else, the packet is discarded.

The main improvement over CSR Mesh is the scalability: this mesh network was deployed using 3 CSR devices and 9 final devices (8 sensor devices and 1 BLE controller). It increases the network sustainability and reduces the cost (to deploy a network with 200 sensor devices is not necessary to use 200 bridge devices). In addition, the total coverage range of network is maintained, since the number of devices which retransmit the packets is the same.

As seen before, the CSR devices retransmit each received packet 3 times by default. Although this number can be reduced, the network PLR is increased. For this reason, our mesh proposal has been evaluated using both CSR device configurations.

#### 7.2.1. CSR Devices Retransmit Each Packet Three Times: Default Mode

In this evaluation, CSR devices were configured in default mode, while Waspmote devices were using the two different configurations described before: *Low Network Load* (each packet is transmitted only once by Waspmote devices) and *High Network Load* (each packet is transmitted three times by Waspmote devices).

Table 7 and Table 8 show the received and sent packets, respectively, by different devices in this study. Specifically, Table 7 shows the number of received packets by CSR devices and by BLE server. As said before, CSR devices process the received packet, and retransmit it only the first time it is received (the repeated packets are discarded). However, the CSR API is not completely open, and to know the number of retransmitted packets is not possible. Results show an increase of the number of received packets in the *High Network Load* configuration, due to the high number of original packets from each sensor node (see Table 8) and the number of repetitions for each original packet (three instead of one). In this case, the node with the highest number of received packets is CSR2, due to its placement (see Figure 14).

Table 8 shows the number of original packets sent by sensor nodes for each network configuration (*Low Network Load* and *High Network Load*). In this case, sensor nodes sent broadcast packets to all devices in their coverage range, so that, it is not possible to know the receiver device. Despite of this fact, all sensor nodes sent a similar number of packets for each configuration, being received a higher number of packets for the second configuration.

**Figure 15:**
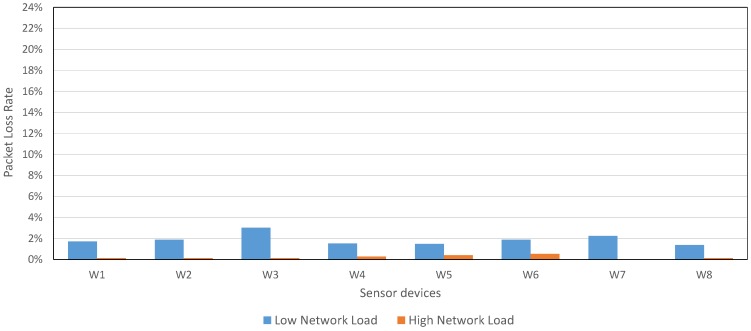
PLR in our mesh proposal for 8 sensor devices with *Individual Mesh* configuration and 3 CSR devices with default configuration.

As Figure 15 shows, even using a *Low Network Load*, the PLR average (1.89%) was lower than the PLR average from CSR Mesh (around 3%) using the recommended configuration. In addition, the network deployed to evaluate our proposal had 8 sensor devices, compared with the 2 sensor devices used in CSR Mesh (is not possible to include a higher number of sensor devices in CSR Mesh due to the bridge restriction).

Moreover, Figure 15 shows the PLR for *High Network Load* configuration: if Waspmote devices transmit each packet 3 times, the network PLR is, on average, only the 0.21%.

Another important parameter to be considered is the network traffic, specially in mesh topologies. Table 9 shows the packets received per second in CSR devices. As it can be seen, even though using the *High Network Load* configuration, the number of packet received by CSR devices was 8.338 on average. In CSR Mesh, we obtained 1.61 packets per second in CSR default mode, but number of sensor devices was only 2. In addition, using the *Low Network Load*, a lower number of packets per second is received by CSR devices (6.564 on average), reducing the traffic network.

In this study, we can see the advantages of our mesh proposal with regard to CSR Mesh topology: our mesh proposal increases the network scalability, and reduces considerably the costs, because the use of CSR bridge devices is not necessary. Moreover, the network PLR has been reduced, and the network traffic is maintained.

Finally, it is possible to reduce further the traffic network, reducing the number of packet retransmitted by CSR devices. Although this measure reduces the network PLR (the default CSR device configuration is modified), to evaluate the behaviour of this configuration in a real environment is interesting. Therefore, next section shows the evaluation for *Individual Mesh* configuration, reducing the number of packet retransmitted by CSR devices.

#### 7.2.2. CSR Devices Retransmit Each Packet Once

In this experiment, CSR devices were configured in a saving mode, retransmitting each received packet once, while Waspmote devices used both two available configurations in our mesh proposal: *Low Network Load* (each packet is transmitted only once by Waspmote devices) and *High Network Load* (each packet is transmitted three times by Waspmote devices).

Table 10 and Table 11 contain data relating to network traffic. Specifically, Table 10 shows the number of packets received by CSR devices, as well as the number of original packets received by the BLE server. As we can see, the highest number of packets is received by CSR2 and CSR3 devices (especially CSR2) for both configurations, due to sensor nodes placement (see Figure 14). Analysing each configuration, an important increase in the number of packets is observed for *High Network Load* configuration (198% for the CSR devices, on average, compared to *Low Network Load*), although the number of packets received by the BLE server is increase only a 12.4%.

Table 11 shows the number of packets sent by each sensor node. Although knowing the immediate destination node is not possible due to the broadcast topology, the number of packets received in the final destination node (BLE server) is showed. In this case, the number of received packets by BLE server is increased when the *High Network Load* configuration is used, as we will analyse later.

**Figure 16:**
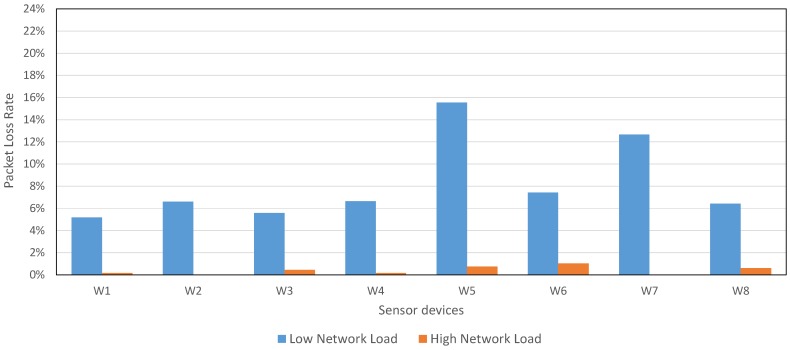
PLR in our mesh proposal for 8 sensor devices with *Individual Mesh* configuration and 3 CSR devices with saving configuration.

As Figure 16 shows, the network PLR average was lower for these configurations, compared with the PLR average from CSR Mesh when CSR devices retransmit each packet once: 8.25% for *Low Network Load* and 0.39% for *High Network Load*, compared with the 16.73% for CSR Mesh retransmitting each packet once. The PLR for *High Network Load* is notable, specially for Industry 4.0 requirements.

Table 12 shows the traffic network, measured in packets per second received in CSR devices. As seen in CSR Mesh experiment, when packets were retransmitted only once, 1.04 packets were received in each CSR device, on average, for two sensor devices. Using our mesh proposal, we achieved a 2.71 packets per second received by each CSR device, on average, for 8 sensor devices configured with a *Low Network Load*, which guarantees the network scalability. Moreover, to minimize the PLR is possible using *High Network Load* configuration, although network traffic is slightly higher (5.19 on average for 8 sensor devices).

In this experiment, our proposal achieved a lower PLR than CSR Mesh; although the network traffic was increased, given that there was a higher number of sensor devices. However, the PLR for this configuration was higher than the PLR achieved for the previous configuration (CSR devices in default mode).

In these experiments, the *Individual Mesh* configuration was evaluated. However, our mesh proposal has another configuration: *Collaborative Mesh*. In this configuration, every device on the network collaborates to create the mesh network, retransmitting the received packets. Next section details this configuration evaluation.

### 7.3. Collaborative Mesh Evaluation

In this second configuration, all devices collaborate to create the mesh network. For this reason, this configuration provides a greater scalability, due to allowing, even, deploying a mesh network using any BLE device or only sensor devices. In addition, this configuration increases the coverage range of the network, because the number of devices which retransmits packets is higher, maintaining the network cost (specific mesh devices are not necessary). This advantage is particularly important when mobile devices are included, because the network coverage range varies in a dynamic way.

It is important to highlight that this configuration of our topology can not be implemented in the CSR Mesh topology, due to two reasons: first, the restriction of adding new devices using CSR devices as bridges creates the need for adding a new CSR device for each new device; second, this requirement limits the role of the new devices to master.

As the previous experiment, two different configurations for CSR devices are used to evaluate the behaviour of this collaborative mesh in both cases: default mode and saving mode.

#### 7.3.1. CSR Devices Retransmit Each Packet Three Times: Default Mode

In this experiment, CSR devices were configured in default mode: retransmitting each received packet three times. Two available configurations were used for Waspmote devices: *Low Network Load* (each packet is transmitted once by Waspmote devices, and the received packets in these packets are retransmitted once the first time that they are received) and *High Network Load* (each packet is transmitted, or retransmitted if it is received, three times).

Table 13 shows the number of packets received by CSR devices which make up the core of this mesh network. The number of packets received is increased, compared with the *Individual Mesh*, due to the fact that in this *Collaborative Mesh* all devices collaborate for retransmitting packets and extending the network coverage range. In both cases, CSR2 has received the highest number of packets, due to its placement (see Figure 14), as in *Individual Mesh* configuration. Further investigations regarding this lack of network load balance are required.

Table 14 shows the number of packets sent by sensor nodes and the number of these packets received by the final device: the BLE server. In this case, the number of retransmitted packets has also been included, owing to sensor nodes also retransmit packets in this *Collaborative Mesh*, as explained above.

**Figure 17:**
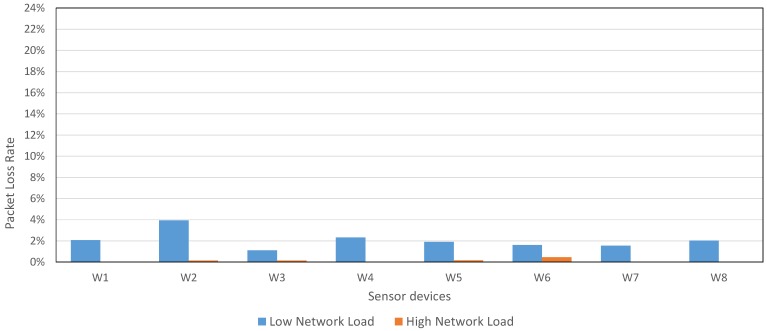
PLR in Collaborative Mesh for 8 sensor devices and 3 CSR devices with default configuration.

Figure 17 shows the PLR for each Waspmote device using both configurations, *Low Network Load* and *High Network Load*. The PLR for Waspmote devices is, on average, 2.06% for *Low Network Load* and 0.11% for *High Network Load*. This network PLR is lower than CSR Mesh PLR, although the number of sensor devices was 8 in this experiment and 2 in CSR Mesh topology experiment.

Comparing this configuration with *Individual Mesh* configuration, whose PLR is 1.89% and 0.21% (for *Low Network Load* and *High Network Load*, respectively), there is not a great difference. However, the main contrast is that this configuration allows increasing the coverage range of the network, due to all devices collaborate to retransmit the packets.

Table 15 shows the traffic network, measured in packet per second received in CSR devices. As seen before, using CSR Mesh configuration, devices received, on average, 1.61 packets per second when packets were retransmitted three times, for two sensor devices. In this experiment, the number of packets received was higher (8.44 for *Low Network Load* and 11.76 for *High Network Load*), but also the number of sensor devices, which is 8 in this case.

This configuration keeps the advantages of our proposal: its a PLR is close to zero, and increases the scalability of network regarding to CSR Mesh. Moreover, compared with the *Individual Mesh* configuration, the coverage area is increased, although the traffic network for this configuration is slightly higher.

Finally, the next section details the evaluation of the behaviour of this configuration in a real environment, using the saving mode for CSR devices.

#### 7.3.2. CSR Retransmit Each Packet Once

In this experiment, CSR devices were configured to retransmit each received packet once, while Waspmote devices used two different configurations described before: *Low Network Load* and *High Network Load*.

Table 16 shows the number of packets received by CSR devices (the core of our mesh network) and by the BLE server. As results shows, for *Low Network Load*, the number of packets received is lower than the number of packets received when CSR devices were configured in default mode. It is expected since, with the current configuration, CSR devices repeat each packet less times. In addition, for *High Network Load*, the number of packets received by CSR devices is greatly increased, although the number of packets (sent and retransmitted by sensor nodes) does not increase (see Table 17). This is due to the used configuration, which triples the number of repetitions of this packets. However, these numbers are very high compared with the collaborative mesh with CSR devices configured in default mode (see Table 13. In future work, a study will be conducted to understand this fact.

Table 16 shows the number of packets sent by sensor nodes, the number of original packets from each sensor node received by BLE server and also the number of packets retransmitted by each sensor node. The highest number of original packets is obtained for the *High Network Load* configuration.

**Figure 18:**
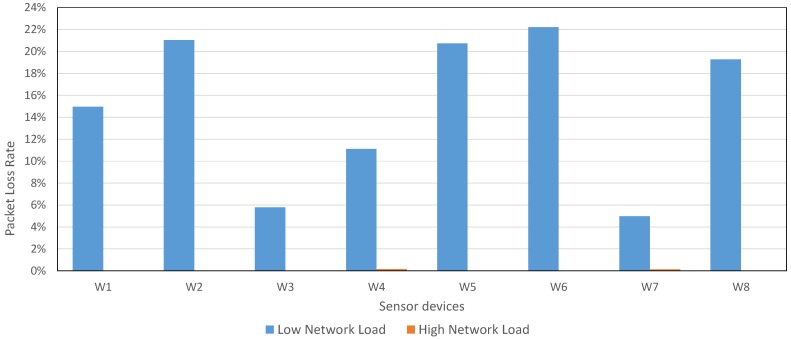
PLR in Collaborative Mesh for 8 sensor devices and 3 CSR devices with saving mode configuration.

Figure 18 shows the PLR for both Waspmote configurations. As shown, the PLR for *Low Network Load* is around 15% on average, too high for our *Zero fails*. It is the highest of all configurations for our mesh proposal, and similar to the PLR obtained for 2 sensor devices by CSR Mesh topology, using the saving mode configuration in CSR devices. However, the PLR for *High Network Load* is 0.03%, the lowest of all configuration, including CSR Mesh.

Table 18 shows the network traffic. For *Low Network Load*, the traffic network is low for 8 sensor devices. However, its PLR is too high for real network requirement. For *High Network Load*, the traffic network is very high. This high traffic network explains the low PLR.

As can be seen, *Low Network Load* for Waspmote devices with CSR devices in saving mode is not a good option for Industry 4.0 due to its *Zero fails* requirement. However, *High Network Load* achieved an incredible PLR, but a higher traffic network. As always in networks, we have to evaluate the importance and priority of these parameters. For our aim, the most important parameters are: to get a PLR as close to zero as possible and to provide a wide network coverage, both with an acceptable network traffic which is not increased in a huge way compared with the traffic on CSR Mesh network.

## 8. Conclusions

This paper has introduced the need of using a new BLE topology which fulfills the requirements in the new Industry 4.0. Firstly, existing mesh topologies have been documented. Secondly, we have chosen CSR Mesh since it allows us to take advantage of the use of BLE 4.1 radio chips, which are not very common in devices intended for IoT and Industry 4.0 use.

In addition, we have proposed a new mesh topology, with different configuration modes, which has been evaluated for different use cases in an emulated Industry 4.0 environment. Our topology allows us to obtain the following benefits with respect to CSR Mesh protocol:

- To reduce the PLR (improving the network performance). Our proposal has obtained a lower PLR for sensor devices. Even in networks with a higher number of sensor devices, several configurations of our proposal maintain this rate close to zero fails required in Industry 4.0.
- To increase the coverage area of the network. All devices can retransmit packets received from the mesh ensuring the total coverage (another Industry 4.0 requirement).
- To increase the scalability of the network. It is achieved by allowing any device with a BLE chip to be part of the mesh without master-slave connection.
- To improve the user experience. The most common user devices that will use this mesh network are mobile devices (for example, smartphones or smartbands), which can communicate with all the devices of the mesh without reconnecting each time when the user leaves the coverage area of his bridge device.
- To provide an open packet format for BLE mesh topology.

Moreover, results obtained for different network configurations show that each configuration works better in a particular use case:

- *Collaborative Mesh* for use cases in which to cover totally an area using the minimum number of devices is needed.
- *Individual Mesh* for use cases where the number of network devices is superior to the number of devices needed to cover totally the area. Both configurations can be combined to create an hybrid network, in which some devices use a configuration while the rest of them use the other one.

Regarding the number of packets per second, we can select in a flexible way the best configuration for our network topology, according to the particular use case: *Individual Mesh* configurations for small spaces with a lot of sensor devices and *Collaborative Mesh* for large areas due to it increases the network coverage with a low cost. Another possible option is to combine both configuration in the same network. However, this is not possible in the evaluated CSR topology, since it has a fixed configuration, and it also requires a bridge device for each new device in the network.

Although the configurations proposed in this paper (*Collaborative Mesh* and *Individual Mesh*) use broadcast transmissions like CSR Mesh, there are several differences between them. The main differences between CSR Mesh protocol, and the *Individual Mesh* and the *Collaborative Mesh* proposed are showed in Table 19.

Despite the advantages of BLE, due to network requirements and a lack of standard for mesh topology (not yet available in last BLE version [30]), different alternatives are needed. Our proposal provides an open protocol, built over BLE and completely compatible with it (in contrast to CSR Mesh [27]). In addition, our proposal uses the broadcast transmission mode, which allows us to take advantage over other alternatives that uses master-slave connections (see [28]), like a rapid network establishment, a greatly device mobility and a greater reliability (the failure of a central device will not disconnect all attached devices). However, there are some shortcomings in our proposal, which are given by the type of data transmission, like security (packets are coded but not encrypted), and the impossibility of ensuring that a packet is received in its destination node, because there are no mechanisms implemented for that at this time. Future works regarding these weaknesses are required.

In conclusion, our contribution regarding with Industry 4.0 requirements is summarised below:

- Zero fails: our proposal improves the CSR Mesh PLR. In addition, the broadcast transmissions make our proposed mesh more flexible and reliable (a device failure does not cause a network failure).
- Sustainability: the number of network devices in our mesh topology has been reduced in comparison with CSR Mesh, due to the use of bridge devices is not needed for new devices. For example, for a network with 8 sensor nodes and a BLE server we have used 3 CSR devices, while 9 CSR devices are needed as bridge devices in CSR Mesh.
- Total coverage: in our proposal, all devices collaborate to retransmit mesh packets, increasing the network coverage range with the same number of devices. In addition, when mobile nodes are included, this coverage range will be total and dynamic, depending on users movement.

Finally, there are still some points on which we must continue working:

- To reduce the number of duplicated packets, and to ensure the packets are received by implementing some methods like packet priority or ACK mechanism. It will allow us to fulfil our Industry 4.0 aims: zero fails and sustainability.
- To test the mesh network with a higher number of static devices (equipped with sensors).
- To include mobile devices in the network (smartphones, smartbands, smartwatches, etc).
- To include devices with actuators to check the correct transmission for different destinations.
- To allow users to retransmit packets from other devices, varying the network in a dynamic way depending on the users movement and proving total network coverage for all users and devices.
- To improve the collaborative mesh security.
- To use LoRa devices to improve communications by extending the coverage range.

## Figures and Tables

**Table 1:** PLR (%) in data transmission between a single broadcaster and a single CSR observer device, according to the number of packet repetition.

Transmissions of Each Data Packet	Packet Loss Rate (%) for Original Packets
1	16.24
2	2.79
3	0.00

**Table 2:** Average RSSI obtained in different scenarios.

Scenario Description	RSSI with Favourable Antenna Position on Average	RSSI with Unfavourable Antenna Position on Average
Direct Line of Sight	−67	−57
Dividing panels	−65	−63
Doors	−68	−73
Columns	−80	−83
Walls	−60	−62
Walls + storage racks	−79	−95
Glass	−65	−70

**Table 3:** Packets sent by sensor nodes and received by BLE server, for each CSR devices configuration.

Sensor Nodes	CSR Devices Transmit Each Packet Once		CSR Devices Transmit Each Packet Three Times	
	Sent Packets	Packets Received by Server	Sent Packets	Packets Received by Server
W1	555 (to CSR2, master-slave connection)	554	577 (to CSR2, master-slave connection)	575
W2	551 (to CSR3, master-slave connection)	367	577 (to CSR3, master-slave connection)	542

**Table 4:** Packets received by CSR devices and BLE server in CSR Mesh evaluation, for each CSR device configuration.

Configuration	Packets Received by			
	CSR1	CSR2	CSR3	Server
CSR devices transmit each packet once	2186	4957	3778	921
CSR devices transmit each packet three times	3629	5542	6003	1117

**Table 5:** PLR for 2 sensor devices using the CSR mesh topology, for different CSR device configurations.

Configuration	Packet Loss Rate (%)	
	Waspmote 1	Waspmote 2
CSR devices transmit each packet once	0.18	33.39
CSR devices transmit each packet three times	0.35	6.07

**Table 6:** Packets per second received by CSR devices in a network with 2 sensor devices and CSR mesh topology.

Configuration	Packets Received per Second			
	CSR1	CSR2	CSR3	Average
CSR devices transmit each packet one	0.62	1.41	1.08	1.04
CSR devices transmit each packet three times	1.16	1.76	1.91	1.61

**Table 7:** Number of packets received by CSR devices and BLE server for each Waspmote device configuration in Individual Mesh with CSR devices retransmit each packet three times.

Number of Packets Transmitted by Waspmote Devices	Packets Received by			
	CSR1	CSR2	CSR3	Server
1 (Low Network Load)	15,587	26,306	21,618	4761
3 (High Network Load)	23,950	43,306	32,070	6132

**Table 8:** Number of packets sent by each sensor node and received by BLE server, for each Waspmote device configuration in Individual Mesh with CSR devices retransmit each packet three times.

Sensor Nodes	Low Network Load		High Network Load	
	Sent Packets	Packets Received by BLE Server	Sent Pakcets	Packets Received by BLE Server
W1	644	633	781	780
W2	582	571	811	810
W3	594	576	745	744
W4	588	579	748	746
W5	607	598	763	760
W6	635	623	768	764
W7	627	613	747	747
W8	576	568	782	781

**Table 9:** Packets per second received by CSR devices when they retransmit each packet 3 times (default mode). Waspmote devices configured as *Individual Mesh*.

Number of Packets Transmitted by Waspmote Devices	Packets Received per Second			
	CSR1	CSR2	CSR3	Average
1 (Low Network Load)	4.833	8.157	6.703	6.564
3 (High Network Load)	6.031	10.906	8.076	8.338

**Table 10:** Number of packets received by CSR devices and BLE server for each Waspmote device configuration in Individual Mesh with CSR devices retransmit each packet once.

Number of Packets Transmitted by Waspmote Devices	Packets Received by			
	CSR1	CSR2	CSR3	Server
1 (Low Network Load)	7172	11,481	9197	4814
3 (High Network Load)	15,628	20,480	18,395	5410

**Table 11:** Number of packets sent by each sensor node and received by BLE server, for each Waspmote device configuration in Individual Mesh with CSR devices retransmit each packet once.

Sensor Nodes	Low Network Load		High Network Load	
	Sent Packets	Packets Received by BLE Server	Sent Pakcets	Packets Received by BLE Server
W1	677	642	669	668
W2	668	624	695	695
W3	646	610	681	678
W4	632	590	670	669
W5	637	538	684	679
W6	660	611	682	675
W7	672	587	681	681
W8	654	612	669	665

**Table 12:** Packets per second received by CSR devices when they retransmit each packet once. Waspmote devices configured as *Individual Mesh* configuration.

Number of Packets Transmitted by Waspmote Devices	Packets Received per Second			
	CSR1	CSR2	CSR3	Average
1 (Low Network Load)	2.096	3.356	2.688	2.714
3 (High Network Load)	4.469	5.856	5.260	5.195

**Table 13:** Number of packets received by CSR devices and BLE server for each Waspmote device configuration in Collaborative Mesh with CSR devices retransmit each packet three times.

Number of Packets Transmitted by Waspmote Devices	Packets Received by			
	CSR1	CSR2	CSR3	Server
1 (Low Network Load)	36,917	49,824	34,377	6326
3 (High Network Load)	51,316	77,035	63,009	5411

**Table 14:** Number of packets sent and retransmit by each sensor node and received by BLE server, for each Waspmote device configuration in Collaborative Mesh with CSR devices retransmit each packet three times.

Sensor Nodes	Low Network Load			High Network Load		
	Sent Packets	Packets Received by BLE Server	Retransmitted Packets	Sent Pakcets	Packets Received by BLE Server	Retransmitted Packets
W1	817	800	2928	709	709	4334
W2	787	756	2889	690	689	2143
W3	816	807	3076	688	687	1332
W4	814	795	3101	705	705	939
W5	787	772	3128	626	625	3211
W6	811	798	3148	655	652	4054
W7	838	825	3181	678	678	4354
W8	789	773	2882	666	666	4090

**Table 15:** Packet per second received by CSR devices when they retransmit each packet 3 times (default mode). Waspmote devices configured as Collaborative Mesh.

Number of Packets Transmitted and Retransmitted by Waspmote Devices	Packets Received per Second			
	CSR1	CSR2	CSR3	Average
1 (Low Network Load)	7.720	10.419	7.189	8.443
3 (High Network Load)	9.464	14.208	11.621	11.764

**Table 16:** Number of packets received by CSR devices and BLE server for each Waspmote device configuration in Collaborative Mesh with CSR devices retransmit each packet once.

Number of Packets Transmitted by Waspmote Devices	Packets Received by			
	CSR1	CSR2	CSR3	Server
1 (Low Network Load)	10,384	14,700	12,589	4617
3 (High Network Load)	95,138	116,130	100,465	5218

**Table 17:** Number of packets sent by each sensor node and received by BLE server, for each Waspmote device configuration in Collaborative Mesh and CSR devices retransmit each packet once.

Sensor Nodes	Low Network Load			High Network Load		
	Sent Packets	Packets Received by BLE Server	Retransmitted Packets	Sent Pakcets	Packets Received by BLE Server	Retransmitted Packets
W1	695	591	3006	652	652	2081
W2	680	537	2997	638	638	2058
W3	690	650	3089	660	660	2161
W4	666	592	2976	660	659	2176
W5	690	547	3046	641	641	2115
W6	680	529	2979	643	643	2121
W7	683	652	3037	670	669	2199
W8	643	519	2921	656	656	2069

**Table 18:** Packets per second received by CSR devices when they retransmit each packet once. Waspmote devices configured as Collaborative Mesh.

Number of Packets Transmitted and Retransmitted by Waspmote Devices	Packets Received per Second			
	CSR1	CSR2	CSR3	Average
1 (Low Network Load)	2.833	4.010	3.434	3.425
3 (High Network Load)	25.316	30.902	26.734	27.651

**Table 19:** Differences between CSR Mesh protocol, *Individual Mesh* and *Collaborative Mesh*.

Evaluated Characteristics	CSR Mesh	Individual Mesh	Collaborative Mesh
Open protocol	No	Yes	Yes
Bridge device needed to use the network	Yes	No	No
Master-Slave connections	Among new devices and bridge devices	No	No
Necessary BLE version	4.1 or higher for bridges 4.0 or higher for rest of nodes	4.0 or higher	4.0 or higher
All devices retransmit mesh packets	No	No	Yes

## Abbreviations

The following abbreviations are used in this manuscript:

I3A	Albacete Research Institute of Informatics
WSN	Wireless Sensor Network
RSSI	Received Signal Strength Indicator
PLR	Packet Loss Rate
IoT	Internet Of Things
BLE	Bluetooth Low Energy
CSR	Cambridge Silion Radio
TTL	Time To Live
PDU	Payload Data Unit
CRC	Cyclic Redundancy Check
